# Polyclonal Recipient nTregs Are Superior to Donor or Third-Party Tregs in the Induction of Transplantation Tolerance

**DOI:** 10.1155/2015/562935

**Published:** 2015-07-27

**Authors:** Nina Pilat, Christoph Klaus, Karin Hock, Ulrike Baranyi, Lukas Unger, Benedikt Mahr, Andreas M. Farkas, Fritz Wrba, Thomas Wekerle

**Affiliations:** ^1^Section of Transplantation Immunology, Department of Surgery, Medical University of Vienna, 1090 Vienna, Austria; ^2^Institute of Clinical Pathology, Medical University of Vienna, 1090 Vienna, Austria

## Abstract

Induction of donor-specific tolerance is still considered as the “Holy Grail” in transplantation medicine. The mixed chimerism approach is virtually the only tolerance approach that was successfully translated into the clinical setting. We have previously reported successful induction of chimerism and tolerance using cell therapy with recipient T regulatory cells (Tregs) to avoid cytotoxic recipient treatment. Treg therapy is limited by the availability of cells as large-scale expansion is time-consuming and associated with the risk of contamination with effector cells. Using a costimulation-blockade based bone marrow (BM) transplantation (BMT) model with Treg therapy instead of cytoreductive recipient treatment we aimed to determine the most potent Treg population for clinical translation. Here we show that CD4^+^CD25^+^ *in vitro* activated nTregs are superior to TGF*β* induced iTregs in promoting the induction of chimerism and tolerance. Therapy with nTregs (but not iTregs) led to multilineage chimerism and donor-specific tolerance in mice receiving as few as 0.5 × 10^6^ cells. Moreover, we show that only recipient Tregs, but not donor or third-party Tregs, had a beneficial effect on BM engraftment at the tested doses. Thus, recipient-type nTregs significantly improve chimerism and tolerance and might be the most potent Treg population for translation into the clinical setting.

## 1. Introduction

Solid organ transplantation is the only curative treatment for many end-stage organ diseases and although short-term survival rates have improved remarkably during the last decades, long-term outcome is still limited [1]. Current immunosuppressive therapies (mostly relying on calcineurin inhibitors) have proven to be exceptionally potent in the prevention of acute rejection episodes; however late graft loss due to chronic rejection is still a major problem [2] and chronic immunosuppressive treatment causes substantial morbidity and mortality. The induction of donor-specific immunological tolerance would obviate the need for life-long immunosuppressive therapy in organ transplant recipients while eliminating the risk of chronic rejection. Unlike many other tolerance regimens, the mixed chimerism approach has been successfully translated into the clinical setting [3]; widespread clinical application however is impeded by the toxicity of current BMT protocols [4]. Despite the advancements in the development of nonmyeloablative, so-called reduced conditioning protocols, conditioning-related toxicities and graft-versus-host disease (GVHD) are still major problems in human BMT, especially across HLA barriers [5]. The goal of establishing noncytoreductive mixed chimerism protocols to induce transplantation tolerance has been in focus of mixed chimerism research for decades [4], leading to stepwise development of minimum conditioning regimens. The administration of costimulation blockers allowed the development of nonmyeloablative protocols that are devoid of global T cell depletion; however the need for cytoreduction (by either irradiation or cytotoxic drugs) could only be overcome by the use of “mega” doses of BM (~200 × 10^6^ cells/mouse → 1 × 10^10^ cells/kg), which is not realistic for clinical translation [6, 7].

Recently, we developed a BMT protocol that is devoid of cytoreductive recipient treatment, while using clinically realistic doses of BM (~15–20 × 10^6^ cells/mouse → 7.5–20 × 10^8^ cells/kg) [8, 9], by combining therapeutic administration of Tregs and costimulation blockade [10, 11]. Treg treatment not only facilitates BM engraftment without the need for irradiation or cytotoxic drugs, but also prevents chronic rejection of donor cardiac grafts [12]. For translation into nonhuman primate models or into the clinical setting, it would be desirable to know which Treg population is preferable in terms of efficacy and safety. Moreover, a dose titration is necessary in order to find the optimal/minimal dose to attain a therapeutic effect. Previously, we tested different populations of recipient-type Tregs for their therapeutic potential to promote BM engraftment. Polyclonal FoxP3 Tregs were produced by retroviral transduction of wild-type B6 CD4^+^ lymphocytes with a retroviral vector containing FoxP3 [10, 13], but although large numbers can be generated for experimental purposes, the role of FoxP3 in human Tregs is more complex [14] and retroviral transduction implicates the risk of insertional mutagenesis secondary to gene insertion into the host chromosome, which could lead to disruption or activation of cellular genes. CD4^+^CD25^+^ nTregs were sorted from B6 spleen and lymph nodes and cultured* in vitro* with the purpose of activation, but due to their rarity, sufficient cell numbers pose a problem in both experimental and clinical setting. For experimental purposes, generation of induced Tregs by* in vitro* culture in the presence of TGF*β* and IL2 is an attractive alternative as it allows the production of large quantities of Tregs [15, 16]; however this approach is suggested to be less effective in human T cells [17].

We could already show that polyclonal recipient Tregs potently suppress alloreactivity across MHC barriers, preventing the rejection of fully mismatched BM in nonirradiated wild-type hosts [10]. It has been proposed in* in vitro* studies that once activated, the mechanism of suppression by Tregs was proposed to be* nonspecific* and not dependent on TCR engagement [18, 19]. More recently, TCR signaling was shown to be dispensable for FoxP3 expression but a functional TCR is critically required for suppressive function* in vivo* [20]. Although it remains unclear whether already activated (either in the thymus or* in vitro*) Treg needs another TCR stimulus to exert full suppressor potential, an alternative approach for Treg therapy could use CD28-mediated costimulation with a CD28 superagonistic monoclonal antibody to substitute TCR engagement [21]. As in our model, Tregs are already in an activated state after* in vitro* culture; we hypothesized that they do not need an additional stimulus to prevent BM rejection. Therefore, we aimed to determine the effectiveness of donor (Balb/c) and unrelated, third-party (C3H) Tregs with respect to their potency to induce chimerism and tolerance. If third-party (“off-the-shelf”) Tregs are effective, they could be expanded in advance and banked upon use, which would be highly relevant for clinical use.

## 2. Materials and Methods

### 2.1. Animals

Female C57BL/6 (B6, recipient, H-2^b^), Balb/c (donor, H-2^d^), and C3H/HeNCrl (C3H, third party, H-2^k^) mice were purchased from Charles River Laboratories (Sulzfeld, Germany), housed under specific pathogen-free conditions, and used at 6 to 12 weeks of age. All experiments were approved by the local review board of the Medical University of Vienna and the Austrian Federal Ministry of Science, Research and Economy and were performed in accordance with national and international guidelines of laboratory animal care.

### 2.2. BMT Protocols

Groups of age-matched B6 recipients received 15–20 × 10^6^ unseparated BM cells from Balb/c donors by injection into the tail vein and costimulation blockade with anti-CD154 mAb (anti-CD40L, MR1, 1 mg, d0), CTLA4Ig (0.5 mg, d2 and d4), and rapamycin (0.1 mg/mouse, d−1, d0, and d2) [10]. Groups of mice received different doses of nTregs or iTregs (0.1–5 × 10^6^ cells) simultaneously with BMT as indicated.

### 2.3. Generation of Tregs

Tregs were generated as described previously. Shortly, cells were isolated from spleen and lymph nodes of naïve B6 mice. For nTreg generation CD4^+^CD25^+^ cells were purified by magnetic bead separation (CD4^+^CD25^+^ Regulatory T cell Isolation Kit, Miltenyi Biotec) and cultivated for 5 days in precoated plates (anti-CD3, anti-CD28, Biolegend) in the presence of 100 U/mL IL-2 (Sigma). For iTreg generation CD4^+^ cells were isolated (L3T4 microbeads, Miltenyi Biotec) and cultured for 5 days in precoated plates (anti-CD3, anti-CD28) in the presence of 100 U/mL IL-2 and 5 ng/mL rhTGFbeta (R&D Systems) [16]. Purity of MACS sorted populations was >90%. At the end of culture, the Treg enriched cell populations were used for therapeutic intravenous administration without additional sorting steps [10].

### 2.4. Antibodies and Flow Cytometric Analysis

Multicolor flow cytometric analysis of multilineage chimerism was performed as described previously [23, 24]. Briefly, chimerism was calculated as the net percentage of donor MHC class I^+^ (H-2D^d^, 34-2-12) cells among specific leukocyte lineages (CD4^+^ and CD8^+^ T cells, B220^+^ B cells, and Mac1^+^ myeloid cells) [23, 24]. Mice were considered chimeric if donor cells were detectable by flow cytometry within both the myeloid lineage and at least one lymphoid lineage. For analysis of Tregs mAbs with specificity against CD4 (RM4-4) and CD25 (7D4) were used. For intracellular staining a FoxP3 (FJK-16s) staining kit (eBioscience) was used according to the manufacture's protocol. PI was used for dead cell exclusion when appropriate. Surface staining was performed according to standard procedures and flow cytometric analysis was done on Coulter Cytomics FC500 using CXP software (Coulter, Austria) for acquisition and analysis.

### 2.5. Skin Grafting

Full thickness tail skin from donor (Balb/c) and fully mismatched third-party (C3H) mice were grafted 4 to 6 weeks after BMT and visually inspected thereafter at short intervals. Grafts were considered to be rejected when less than 10% remained viable. Grafts that remained viable throughout the follow-up were stored in 4.5% formalin (with a buffered pH of 7.5) and embedded in paraffin within 24 h.

### 2.6. Histological Analysis

4 *μ*m sections were cut from paraffin-embedded tissue, stained with hematoxylin-eosin (HE) and Giemsa according to standard protocols, and analyzed by an experienced pathologist in blinded fashion. Skin allografts were scored according to Banff 2007 working classification of skin-containing composite tissue allograft pathology [22].

### 2.7. Mixed Lymphocyte Reaction (MLR)

MLRs were performed as described in detail previously [6, 10]. Briefly, 4 × 10^5^ responder splenocytes were incubated in triplicate with 4 × 10^5^ irradiated (30 Gy) stimulator cells of either B6 (recipient), Balb/c (donor), or C3H (3rd party) origin or with medium only. After 72 h of incubation, cells were pulsed with [3H]-thymidine (Amersham, Biosciences, UK) for 18 h. Incorporated radioactivity was measured using scintillation fluid in a *β*-counter. Stimulation indices (SI) were calculated in relation to medium controls. Results represent averaged data of triplets from pooled animals.

### 2.8. Anti-Donor Antibodies

Recipient serum harvested >3 months after BMT was heat-inactivated and incubated with recipient-type and donor-type thymocytes. Binding of serum IgG Abs to thymocytes was analyzed by flow cytometry using FITC-conjugated rat anti-mouse IgG1 and IgG2a/2b (BD Pharmingen).

### 2.9. Statistics

A two-sided Student's *t*-test with unequal variances was used to compare chimerism levels and SI values between groups. Fisher's exact test was used to compare chimerism rates between groups and rejection scores. Skin allograft survival was calculated according to the Kaplan-Meier product limit method and compared between groups using the log-rank test. A *p* value less than 0.05 was considered to be statistically significant.

## 3. Results

### 3.1. nTregs Are More Potent Than iTregs in the Induction of Hematopoietic Chimerism

**Figure 1:**
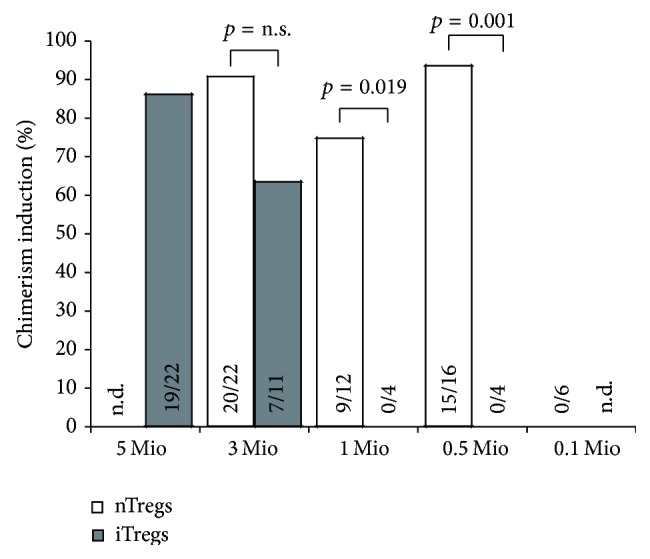
nTregs are superior to iTregs in the induction of mixed chimerism. Groups of B6 mice were grafted with 15–20 × 10^6^ Balb/c BM cells under the cover of costimulation blockade (anti-CD154, CTLA4Ig) and rapamycin and were additionally treated with decreasing numbers of recipient-derived nTregs (white bars) and recipient-derived iTregs (grey bars), respectively. Percentages of successfully induced chimeras are shown. Mice were considered chimeric if donor cells were detectable by flow cytometry within both the myeloid lineage and at least one lymphoid lineage for the length of follow-up. Data are pooled from multiple independent experiments (n.d.: not done).

Recently, we could show that therapeutic administration of polyclonal recipient Tregs enhances BM engraftment and obviates the need for cytoreductive recipient preconditioning. In previous experiments, different Treg populations demonstrated similar suppressive potency* in vitro* and* in vivo* [10]. Cells were used without further sorting at a dose of 3 × 10^6^ cells/mouse for FoxP3 transduced Tregs (FoxP3 Tregs) or* in vitro* activated natural CD4^+^CD25^+^ Tregs (nTregs) [10] and 3–5 × 10^6^ cells/mouse for TGF*β* induced Tregs (iTregs) [10, 12] after* in vitro* culture without further sorting (corresponds to ~150–250 × 10^6^ cells/kg). For nonhuman primate experiments and subsequent clinical application it would be desirable to find the most suitable cell population and the lowest efficient cell number. Therefore, we investigated the potency of nTregs and iTregs in the induction of chimerism and tolerance as we assumed that FoxP3 Tregs would be less suitable for clinical application due to safety issues concerning retroviral transduction [12]. B6 recipients received a conventional dose of fully mismatched Balb/c BM cells under the cover of costimulation blockade (anti-CD154 mAb d0, 1 mg/mouse; CTLA4Ig d2, 0.5 mg/mouse) and short-course rapamycin (d −1/0/+2, 0.5 mg/mouse) combined with decreasing numbers of different Treg populations. Notably, chimerism rates were significantly higher in nTreg than in iTreg treated mice and cell numbers could be reduced to 0.5 × 10^6^ cells/mouse (results were comparable to FoxP3 Tregs, data not shown) (Figure 1).

### 3.2. nTregs Promote Multilineage Chimerism and Donor-Specific Skin Graft Tolerance

**Figure 2:**
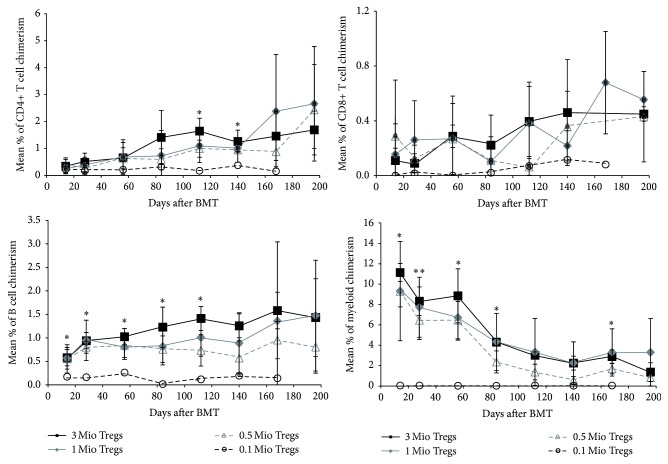
Therapeutic nTreg treatment induces multilineage chimerism at doses of 3, 1, and 0.5 × 10^6^ cells without cytoreduction. B6 BMT recipients were treated with different doses of recipient nTregs (■ 3 × 10^6^ cells, *n* = 4; ◆ 1 × 10^6^ cells, *n* = 8; ∆ 0.5 × 10^6^ cells, *n* = 6; ○ 0.1 × 10^6^ cells, *n* = 6). Donor (H-2D^d+^) chimerism among leukocytes of T cell (CD4^+^ and CD8^+^), B cell (B220^+^), and the myeloid (Mac1^+^) lineage was assessed by flow cytometry of peripheral blood at multiple time points and is shown as mean percent (error bars indicate standard deviation). Data are representative for at least 3 independent experiments per group.

As nTregs have shown to be more potent at lower doses and also reduce the risk of reconversion into effector T cells as they show demethylation of TSDR region, a prerequisite for stable FoxP3 expression and long-term Treg functionality and lineage stability [25, 26], we used this particular Treg population for subsequent experiments. Chimerism in Treg treated BMT recipients was of multilineage nature, with donor populations present in all tested leukocyte lineages, including T cell populations, which is proposed to be a prerequisite for tolerance [27, 28]. Chimerism levels in peripheral blood persisted for the length of follow-up and correlated with chimerism in lymphoid organs (BM and spleen, data not shown). Chimerism levels between BMT recipients that received 3, 1, or 0.5 × 10^6^ cells were comparable without a significant difference; however, BMT recipients that received 0.1 × 10^6^ cells did not develop chimerism in any lineage (*p* < 0.05 for most time points) (Figure 2).

**Figure 3:**
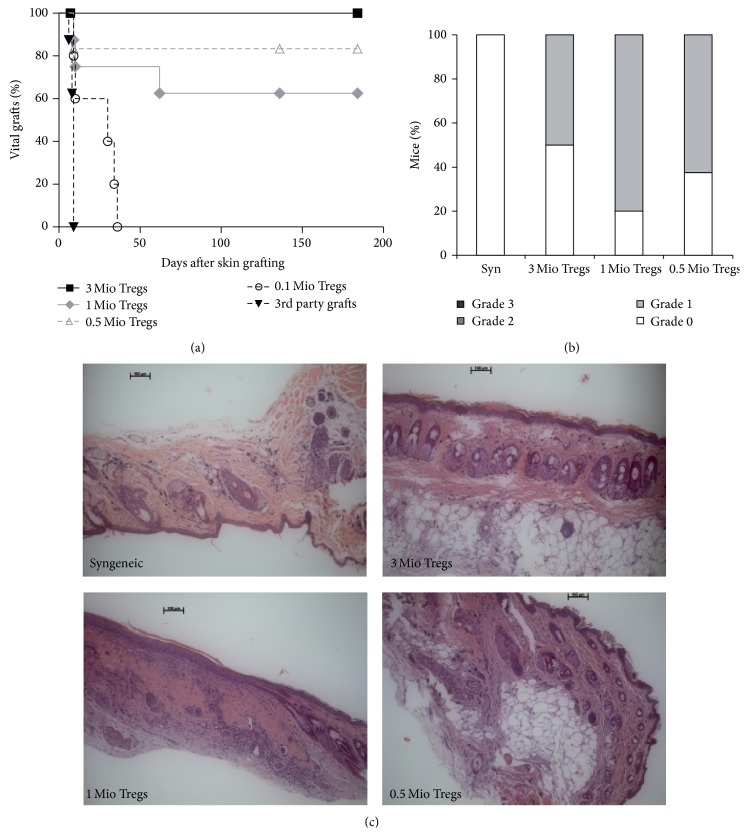
Tolerance is successfully induced in chimeras that received low-dose Treg therapy. Donor-specific tolerance was assessed by grafting of donor and third-party skin 4–6 weeks after BMT. (a) Donor skin graft survival was significantly prolonged in BMT recipients treated with Treg numbers that have been shown to be sufficient to induce chimerism (■ 3 × 10^6^ cells, *n* = 4; ◆  1 × 10^6^ cells, *n* = 8; ∆ 0.5 × 10^6^ cells, *n* = 6;** ○**  0.1 × 10^6^ cells, *n* = 6; ▼  pooled third-party grafts, *n* = 24). Survival was calculated according to the Kaplan-Meier product limit method and compared between groups using the log-rank test (*p* < 0.05 for 0.1 × 10^6^ cells versus each other group). (b) Classification of skin allograft pathology for syngeneic grafts (*n* = 4) and Treg treated groups (3 × 10^6^ cells, *n* = 8; 1 × 10^6^ cells, *n* = 8; 0.5 × 10^6^ cells, *n* = 5; 0.1 × 10^6^ cells, *n* = 8) are shown [22] (Grade 0: no or rare inflammatory infiltrates, skin architecture intact; Grade 1: mild inflammatory infiltration, no involvement of overlying epidermis; Grade 2: moderate perivascular inflammation with mild epidermal/adnexal involvement; Grade 3: severe inflammation, atrophy of epidermis, dyskeratosis, and/or keratinolysis). Data are representative for at least 2 independent experiments per group. (c) Representative histology from skin grafts of Treg treated groups and syngeneic controls (>120 d after skin grafting; HE staining magnification ×100) is shown.

To assess donor-specific tolerance, skin transplants were performed 4 to 8 weeks after BMT. All chimeras induced with Treg doses that successfully developed chimerism (0.5–3 × 10^6^ cells) accepted donor skin for the length of follow-up, whereas mice BMT recipients that failed to develop chimerism uniformly rejected donor skin (Figure 3(a)). Third-party grafts were rapidly rejected in all groups (MST = 9 in all groups, pooled data), indicating immunocompetence in all mice. Histopathologic analysis revealed that grafts of Treg induced chimeras were almost completely free of signs of chronic rejection, regardless of the Treg dose they received, with no significant differences being observed (Figures 3(b) and 3(c)). These data suggest that a dose of 0.5 × 10^6^ nTregs is sufficient for the induction of chimerism and tolerance.

### 3.3. Polyclonal Recipient Tregs Are Superior to Donor and Third-Party Tregs

**Figure 4:**
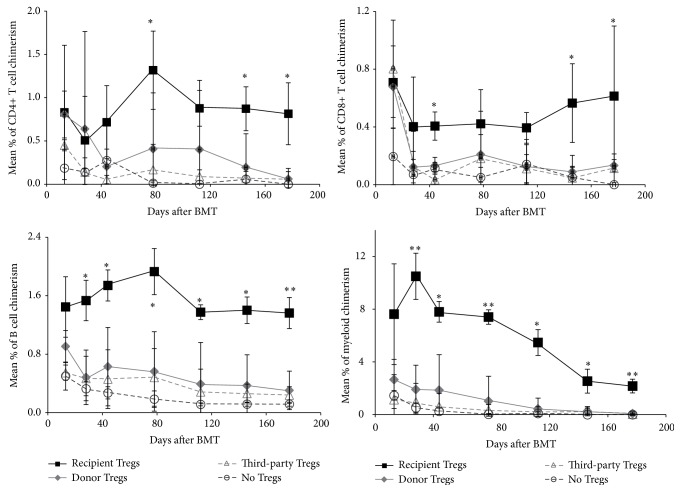
Recipient but not donor and third-party Tregs prevent BM rejection. Groups of B6 mice were grafted with 15–20 × 10^6^ Balb/c BM cells under the cover of costimulation blockade (anti-CD154, CTLA4Ig) and rapamycin (**○** no Tregs; control *n* = 5) and were additionally treated with 3 × 10^6^ nTregs of recipient (■  *n* = 4), donor (◆  *n* = 4), or third-party (∆  *n* = 3) origin. Donor (H-2D^d+^) chimerism among leukocytes of T cell (CD4^+^ and CD8^+^), B cell (B220^+^), and the myeloid (Mac1^+^) lineage was assessed by flow cytometry of peripheral blood at multiple time points and is shown as mean percent (error bars indicate standard deviation). ^*∗∗*^ *p* < 0.005, ^*∗*^ *p* < 0.05 (Student's *t*-test for recipient Tregs versus each other group).

The limited availability of Tregs from a single individual could constitute a major barrier to the implementation of Treg cell-based therapy in the clinical setting. Third-party derived Tregs could be a promising alternative, as they can be prepared and expanded in advance and stored until use. Therefore, we tested the potency of different Tregs sourced to induce chimerism and allograft tolerance.* In vitro* activated nTregs from recipient, donor, or third-party strain were used at a dose of 3 × 10^6^ cells in combination with the Treg BMT protocol [10]. Whereas recipient Tregs again potently induce hematopoietic chimerism, donor and third-party Treg therapy failed to prevent BM rejection. Chimerism in recipient Treg treated mice was permanent and of multilineage nature in all recipients, whereas recipients of donor or third-party Tregs (and controls without Treg treatment) failed to develop multilineage chimerism (*p* < 0.05 for most time points) (Figure 4). Donor Treg treatment led to transient chimerism in one recipient; however, chimerism was restricted to the B cell and myeloid lineages and became undetectable after 4 months after BMT.

**Figure 5:**
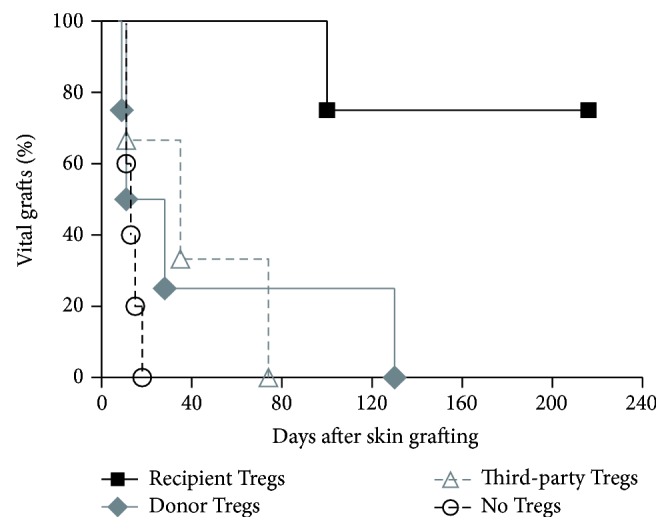
Recipient but not donor and third-party Treg treatment induces donor-specific tolerance. Donor-specific tolerance was assessed by grafting of donor and third-party skin 4–6 weeks after BMT. Donor skin graft survival was significantly prolonged in BMT recipients treated with recipient Tregs (■ recipient Tregs, *n* = 4; ◆ donor Tregs, *n* = 4; ∆ third-party Tregs, *n* = 3;** ○** no Tregs, *n* = 5). Survival was calculated according to the Kaplan-Meier product limit method and compared between groups using the log-rank test (*p* < 0.05 for recipient Tregs versus all other groups).

Assessment of donor-specific tolerance revealed that neither donor nor third-party Tregs were able to significantly prolong skin graft survival (donor Tregs MST = 19.5 days, *p* = 0.256; third-party Tregs MST = 35 days, *p* = 0.0948 versus no Tregs MST = 11 days). Importantly, recipient-type Tregs led to indefinite survival in the majority of recipients (>30 weeks after skin grafting, *p* = 0.0046 versus no Tregs; *p* = 0.0266 versus donor Tregs; *p* = 0.0101 versus third-party Tregs). Skin graft survival was prolonged in one mouse of the donor Treg group, which developed transient chimerism; however it was rejected eventually (Figure 5).

### 3.4. Chimeras Induced through Recipient nTreg Treatment Show Humoral and* In Vitro* Tolerance

**Figure 6:**
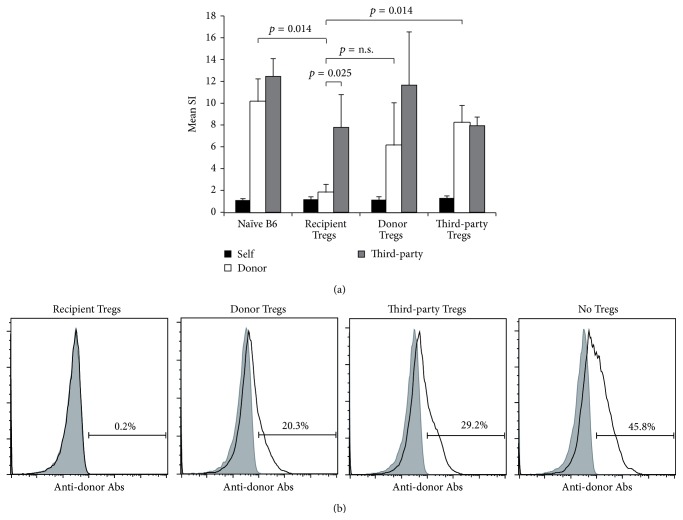
Chimeras induced through recipient Treg treatment demonstrate donor-specific hyporesponsiveness* in vitro* and humoral tolerance. (a) Mixed lymphocyte reaction results from selected BMT recipients were obtained 28–37 weeks after BMT. Chimeras of recipient Treg treated mice (*n* = 4) showed specific hyporesponsiveness to donor antigens* in vitro* (*p* = 0.0247 SI anti-donor compared to third-party antigen; *p* = 0.0183 SI anti-donor compared to naïve B6 mice, *n* = 3). Donor reactivity was preserved in BMT recipients that were treated with donor (*n* = 3) or third-party (*n* = 3) type Tregs. SIs were calculated by dividing the mean cpm from responses against recipient (black column; B6), donor (white column; Balb/c), or third-party (grey column; C3H) stimulator cells by mean background cpm (i.e., cpm with no stimulator population). Error bars indicate standard deviation. (b) BMT recipients were analyzed for the existence of anti-donor antibodies in serum >3 months after BMT (i.e. ~1-2 months after skin grafting). Recipient Treg induced chimeras (*n* = 4) uniformly failed to develop detectable levels of anti-donor antibodies, whereas BMT recipients treated with donor (*n* = 4) or third-party (*n* = 3) Tregs and control mice without Treg treatment (but receiving BM, costimulation blockade, and rapamycin; *n* = 5) developed substantial antibody levels. The reactivity of sera with syngeneic (B6; grey filled area) and donor (Balb/c; black line) thymocytes is shown by flow cytometry through indirect staining with anti-mouse IgG. Representative histograms are shown.

*In vitro* T cell tolerance was also evaluated by performing MLR assays at the end of follow-up (>30 weeks after BMT). Recipient Treg treated chimeras showed specific hyporesponsiveness towards donor antigen* in vitro*, in contrast to BMT recipients treated with donor or third-party Tregs or without Tregs whose response toward donor stimulators was preserved and comparable to naïve mice (reactivity towards donor antigen *p* = 0.014 recipient Tregs versus naïve B6; *p* = 0.014 recipient Tregs versus third-party Tregs). Alloreactivity towards third-party antigens was preserved in all mice, reassuring immunocompetence in chimeras treated with combined BM and Treg cell therapy (recipient Tregs *p* = 0.025 donor versus third-party) (Figure 6(a)). Development of anti-donor Abs (antibodies) was shown to be associated with the development of chronic rejection and late graft loss in clinical transplantation. Moreover it was suggested to be associated with split tolerance or incomplete humoral tolerance in the experimental setting [29]. Serum from BMT recipients was analyzed for the presence of anti-donor antibodies late after BMT and skin grafting (>3 months) through flow cytometric crossmatch. No anti-donor Abs were detectable in chimeras treated with recipient Tregs, whereas BMT recipient treated with donor or third-party Tregs and control mice (BMT recipients receiving the same regimen without Tregs) developed substantial levels of anti-donor Abs (Figure 6(b)). Thus, only Treg therapy with recipient-type cells prevents development of a humoral anti-donor response in BMT recipients.

## 4. Discussion

The study presented demonstrates that polyclonal nTreg therapy prevents rejection of allogeneic BM with higher potency than iTregs, allowing for a 6-fold reduction of Treg dose compared to our previous reports [10]. Moreover these data are evidence that recipient Tregs are superior to donor and third-party Tregs in their capacity to promote BM engraftment, chimerism induction, and tolerance.

The mixed chimerism approach has been successfully translated into the clinical setting; however, widespread clinical application in transplant recipients has been hindered by the toxicity of current BMT protocols. We previously reported a noncytotoxic protocol by combining therapeutic Treg treatment and a clinically feasible number of stem cells. Notably, Treg treatment not only facilitates BM engraftment and the induction of hematopoietic chimerism, but was also shown to prevent chronic rejection of heart allografts, making it superior over protocols based on irradiation or cytostatic drugs [12]. Here, we could show that nTregs are the preferable Treg population for therapeutic application, in both theoretical and quantitative terms. Although generation of iTregs* in vitro* has practical advantages in murine models, as they are easy to obtain in large numbers, the risk of reconversion is not negligible and induction of stable Tregs in human is more complex than in rodents [30]. In our previous reports, all tested Treg populations were effective in promoting the induction of chimerism and tolerance at high doses [10]; however, in dose titration experiments, superiority of nTregs is undisputable. These data suggest that, for clinical trials using cell therapy for mixed chimerism induction, this population might be best suited for both availability of cell numbers and safety concerns. Although it is likely that purity of Treg* in vitro* cultures is never 100%, contamination by T effector cells in nTreg cultures could be further reduced by addition of rapamycin [31]. Likewise, in our experience, contamination by CD25^+^ conventional T cells was less in nTreg than in iTreg cultures.

Although there are several reports questioning the potency of polyclonal Tregs to suppress alloreactivity, there is evidence that at least some suppressor activity is antigen* nonspecific* upon activation [19]; the need for additional TCR signaling in the presence of CD28 costimulation still needs to be determined [20, 21]. Unlike other studies, which needed Tregs specific for (direct and indirectly presented) donor antigens to prevent chronic rejection [32], BMT recipients treated with activated polyclonal recipient Tregs were devoid of signs of chronic rejection (in both skin and heart allografts) in our model. We hypothesize that the absence of cytotoxic recipient conditioning favors the induction of regulatory mechanisms and intragraft tolerance.

Although recipient-type Tregs are used for therapeutic application in most alloimmunity models, donor-type Tregs have been shown to be able to prevent BM rejection [33, 34]. The use of frozen umbilical cord blood (UCB) units for Treg culture and generation of “off-the-shelf” Tregs is an attractive therapeutic approach as T cell subsets are largely naïve and of reduced complexity, enhancing Treg purity by using CD25 as Treg marker [35]. However, when we tried different Treg sources, BM rejection could only be prevented by recipient-derived cells. Although we could not rule out the fact that higher doses of donor or third-party Tregs would also prevent BM rejection, we demonstrated that for mixed chimerism induction recipient cells might be the most suitable Treg source. As banking of infant UCB has increased markedly, it might be an attractive alternative for autologous Treg sources in the future.

In conclusion, we have demonstrated that recipient-type nTregs are the most potent Treg population for the deliberate induction of donor-specific tolerance via the mixed chimerism approach. We think that these data are relevant for the translation to the nonhuman primate setting and clinical trials. Although additional studies are required to reveal detailed mechanisms, the combination of Treg therapy and mixed chimerism might allow widespread clinical application of this powerful tolerance approach in the future.
